# An evaluation of nutrition intervention during radiation therapy in patients with locoregionally advanced nasopharyngeal carcinoma

**DOI:** 10.18632/oncotarget.19381

**Published:** 2017-07-19

**Authors:** Ting Jin, Kai-Xin Li, Pei-Jing Li, Shuang Huang, Xiao-Zhong Chen, Ming Chen, Qiao-Ying Hu, Lei Shi, Yuan-Yuan Chen

**Affiliations:** ^1^ Department of Radiation Oncology, Zhejiang Cancer Hospital, Hangzhou, Zhejiang 310022, People's Republic of China; ^2^ Department of Radiology, Zhejiang Cancer Hospital, Hangzhou, Zhejiang 310022, People's Republic of China; ^3^ Zhejiang Key Laboratory of Radiation Oncology, Hangzhou, Zhejiang 310022, People's Republic of China; ^4^ Department of Radiation Oncology, Quanzhou First Hospital Affiliated to Fujian Medical University, Quanzhou, Fujian 362000, People's Republic of China

**Keywords:** nasopharyngeal carcinoma, radiotherapy, nutrition intervention, weight loss

## Abstract

**Purpose:**

To evaluate the effectiveness of nutrition intervention during radiation for patients with locoregionally advanced (III-IVa) nasopharyngeal carcinoma (NPC).

**Materials and Methods:**

We retrospectively reviewed 117 patients with locoregionally advanced (III-IVa) NPC treated between December 2015 and March 2016 in Zhejiang Cancer Hospital. All the patients underwent radical chemo-radiotherapy. First, all the patients were divided into the nutrition intervention group and the control group, depending on whether they accepted nutrition intervention. Repeated measures were used to analyze the change of nutritional indicators before, during, and after radiation therapy and to simultaneously compare the difference in nutritional status between the two groups at the same time point. Subsequently, the 117 patients were divided into the malnourished group (weight loss > 5%) and the non-malnourished group (weight loss ≤ 5%) according to whether their weight loss was over 5% of their body weight during radiotherapy. Chi-square tests and logistic regression analysis were used to explore the influence factors for the weight loss.

**Results:**

The repeated measures showed that all indicators including weight, body mass index (BMI), albumin, pre-albumin(PA), and prognostic nutritional index (PNI) dramatically declined in both groups compared with their levels before radiation therapy (All *p* < 0.001). However, there was no significant difference between the intervention and non-intervention groups regarding the mean values of nutritional indicators at the same time point, that before, during, and after radiation therapy, except BMI (All *p* > 0.05). Logistic regression analysis revealed grade ≥ 3 radiation-induced oral mucositis as the prognostic factor for a poor nutrition status (odds ratio, OR = 3.232, *p* = 0.021, confidence interval, CI [1.198, 8.820]). Besides this, patients with a decrease of >15% in pre-albumin level were more likely to be malnourished (OR = 2.442, *p* = 0.041, CI [1.036, 5.757]). Similar to that observed in our former analysis, we did not find that existing nutrition intervention can significantly improve nutritional status (OR = 1.217, *p* = 0.704, CI [0.042, 3.348]).

**Conclusions:**

Our study shows that the nutritional status of the patients gradually declined during treatment. We concluded that grade ≥ 3 radiation-induced oral mucositis would aggravate the extent of malnutrition during radiation therapy in patients with locoregionally advanced NPC. Pre-albumin level was a predictive marker for weight loss in patients with NPC. However, current nutrition intervention during radiation therapy can't significantly reverse nutritional status.

## INTRODUCTION

Nasopharyngeal carcinoma (NPC) has an extremely uneven endemic distribution within Southern China and Southeast Asia [1]. In the last two decades, key milestones have been achieved in the treatment of NPC, and there are continual improvements in treatment outcomes. The U.S. National Comprehensive Cancer Network (NCCN) Guidelines recommend concurrent chemoradiotherapy (CCRT) in the presence or absence of adjuvant chemotherapy as the standard treatment for NPCs. Although the benefit of adjuvant chemotherapy is still open to debate, adjuvant chemotherapy is commonly prescribed for patients with locoregionally advanced NPC at our institution and is well tolerated [2, 3]. Significant improvements in the therapeutic effect are achieved with the extensive application of intensity-modulated radiotherapy (IMRT). The addition of concurrent chemotherapy to radiotherapy prolongs the survival of patients with NPC and renders it a controllable and treatable chronic disease. Approximately 76–80% of patients survive for at least 5 years [4–6]. We should therefore pay more attention to the patients’ quality of life (QOL). Three to 50% of patients with head and neck cancer suffer from malnutrition before treatment, and this percentage range of malnourished patients increases to 44–90% during or at the end of radiotherapy [7–12]. Dysphagia, mucositis, nausea, vomiting and xerostomia are common treatment-related problems, however, all these symptoms may result in compromised food intake that can lead to unintentional weight loss or even malnutrition during treatment [12–14]. The prevalence of malnutrition depends on the different definitions used to identify it, and it is associated with lower physical functioning [10], lower immune status [15], chemo(radio)therapy treatment interruption [16], more severe (grade III/IV) radiotherapy-induced toxicity, [17–20] lower quality of life [9, 21–24], and increased mortality [25, 26]. Complications are more frequent in the first three months after radiotherapy [27]. Nutritional therapy in patients with malignant tumors has an important role in multidiscipline therapy. Adequate nutritional care during radiotherapy minimizes weight loss, decreases the impact of the side effects of treatments, and improves quality of life [9, 22, 24, 28–30]. However, very few studies have been conducted worldwide on the nutritional assessment of patients and nutritional therapy for patients with NPCs during radiotherapy. The aim of our study is to evaluate the effectiveness of nutrition intervention during radiation for patients with locoregionally advanced NPC.

## RESULTS

Our study results demonstrated that current nutritional care protocols based on European guidelines [31] was not enough to reduce the risk of undernutrition during radiotherapy in patients with advanced NPC.

The clinical characteristics and treatment information for the 117 patients who met our study criteria were summarized in Tables 1 and 2. All 117 patients included in our analyses had stage III-IVa NPC, and the mean age of the 117 patients was 50.5 years [confidence interval (CI) [48.6, 52.5]]. Ninety one of 117 (78%) patients accepted nutritional support and the remaining 26 did not. The physicians made the decision whether to provide nutritional support by evaluating the patient's nutritional status depending on his or her general condition, food intake, weight, hematology and biochemistry profiles. Of all the patients, including the nutritional support group, almost half of them (50/117, 42.7%) lost more than 5% of their body weight before radiotherapy.

**Table 1:** Baseline characteristics of 117 patients with locoregionally advanced NPC divided by intervention

Characteristics		Non-intervention (*n* = 26, %)	Intervention (*n* = 91, %)	*p* value
Age, mean(SD)	Year	48.8 (12.6)	51.0 (10.3)	0.355
Basic weight, mean(SD)	Kg	66.5 (14.0)	64.9 (11.0)	0.545
Basic BMI, mean(SD)	Kg/m^2^	24.7 (3.4)	23.2 (2.8)	0.026
Weight before RT, mean(SD)	Kg	68.0 (14.6)	65.4 (10.7)	0.423
BMI before RT, mean(SD)	Kg/m^2^	25.2 (3.5)	23.4 (2.7)	0.019
HB before RT, mean(SD)	g/dl	12.7 (1.2)	12.2 (1.4)	0.078
ALB before RT, mean(SD)	g/L	41.2 (3.5)	41.2 (3.2)	0.955
CRP before RT, mean(SD)	mg/L	4.3 (10.9)	4.0 (5.5)	0.844
PA before RT, mean(SD)	mg/L	265.5 (54.9)	272.3 (49.8)	0.550
LDH before RT, mean(SD)	U/L	199.1 (41.7)	205.5 (53.7)	0.577
PNI before RT, mean(SD)		48.4 (4.3)	48.9 (4.4)	0.643
Sex, *n* (%)	Female	10 (38)	23 (25)	0.188
	Male	16 (62)	68 (75)	
T stage, *n* (%)	T1-2	8 (31)	15 (16)	0.106
	T3-4	18 (69)	76 (84)	
N stage, *n* (%)	N0-2	22 (85)	81 (89)	0.543
	N3	4 (15)	10 (11)	
Neo-chemotherapy, *n* (%)	Yes	26 (100)	89 (98)	0.446
	No	0 (0)	2 (2)	
Current chemotherapy, *n* (%)	Yes	25 (96)	89 (98)	0.639
	No	1 (4)	2 (2)	
Radiation technique, *n* (%)	IMRT	21 (81)	72 (79)	0.854
	TOMO	5 (19)	19 (21)	
Weight loss, *n* (%)	≤ 5%	17 (65)	50 (55)	0.343
	**>** 5%	9 (35)	41 (45)	
Mucositis, *n* (%)	0–2	21 (81)	69 (76)	0.598
	≥ 3	5 (19)	22 (24)	

Abbreviations: NPC = nasopharyngeal carcinoma; SD = std. deviation; RT = radiation therapy; BMI = body weight index; HB = hemoglobin; ALB = albumin; CRP = C-reactive protein; PA = pre-albumin; LDH = lactate dehydrogenase; PNI = prognostic nutritional index.

**Table 2:** Baseline characteristics of 117 patients with locoregionally advanced NPC divided by weight loss

Characteristics		Weight loss ≤ 5% (*n* = 67, %)	Weight loss ≤ 5% (*n* = 50, %)	*p* value
Age, mean (SD)	Year	51.7 (10.8)	49.1 (10.8)	0.200
Basic weight, mean (SD)	Kg	64.0 (11.1)	67.0 (12.2)	0.158
Basic BMI, mean (SD)	Kg/m^2^	23.4 (3.1)	23.7 (3.0)	0.703
Weight before RT, mean (SD)	Kg	64.8 (11.5)	67.5 (11.9)	0.231
BMI before RT, mean (SD)	Kg/m^2^	23.8 (3.2)	23.8 (2.8)	0.933
HB before RT, mean (SD)	g/dl	12.2 (1.4)	12.4 (1.4)	0.427
ALB before RT, mean (SD)	g/L	41.2 (3.3)	41.1 (3.2)	0.793
CRP before RT, mean (SD)	mg/L	3.9 (7.6)	4.2 (6.2)	0.817
PA before RT, mean (SD)	mg/L	268.8 (53.8)	273.3 (47.0)	0.640
LDH before RT, mean (SD)	U/L	206.0 (61.9)	201.4 (31.9)	0.608
PNI before RT, mean (SD)		48.8 (4.1)	48.7 (4.6)	0.959
Sex, *n* (%)	Female	23 (34)	10 (20)	0.088
	Male	44 (66)	40 (80)	
T stage, *n* (%)	T1-2	15 (22)	8 (16)	0.390
	T3-4	52 (78)	42 (84)	
N stage, *n* (%)	N0-2	57 (85)	46 (92)	0.254
	N3	10 (15)	4 (8)	
Neo-chemotherapy, *n* (%)	Yes	66 (99)	49 (98)	0.834
	No	1 (1)	1 (2)	
Current chemotherapy, *n* (%)	Yes	65 (97)	49 (98)	0.739
	No	2 (3)	1 (2)	
Radiation technique, *n* (%)	IMRT	56 (84)	37 (74)	0.204
	TOMO	11 (16)	13 (26)	
Intervention, *n* (%)	Yes	50 (75)	41 (82)	0.343
	No	17 (25)	9 (18)	
Mucositis, *n* (%)	0–2	57 (85)	33 (66)	0.015
	≥ 3	10 (15)	17 (34)	

Abbreviations: NPC = nasopharyngeal carcinoma; SD = std. deviation; RT = radiation therapy; HB = hemoglobin; ALB = albumin; CRP = C-reactive protein; PA = pre-albumin; LDH = lactate dehydrogenase; PNI = prognostic nutritional index.

**Figure 1:**
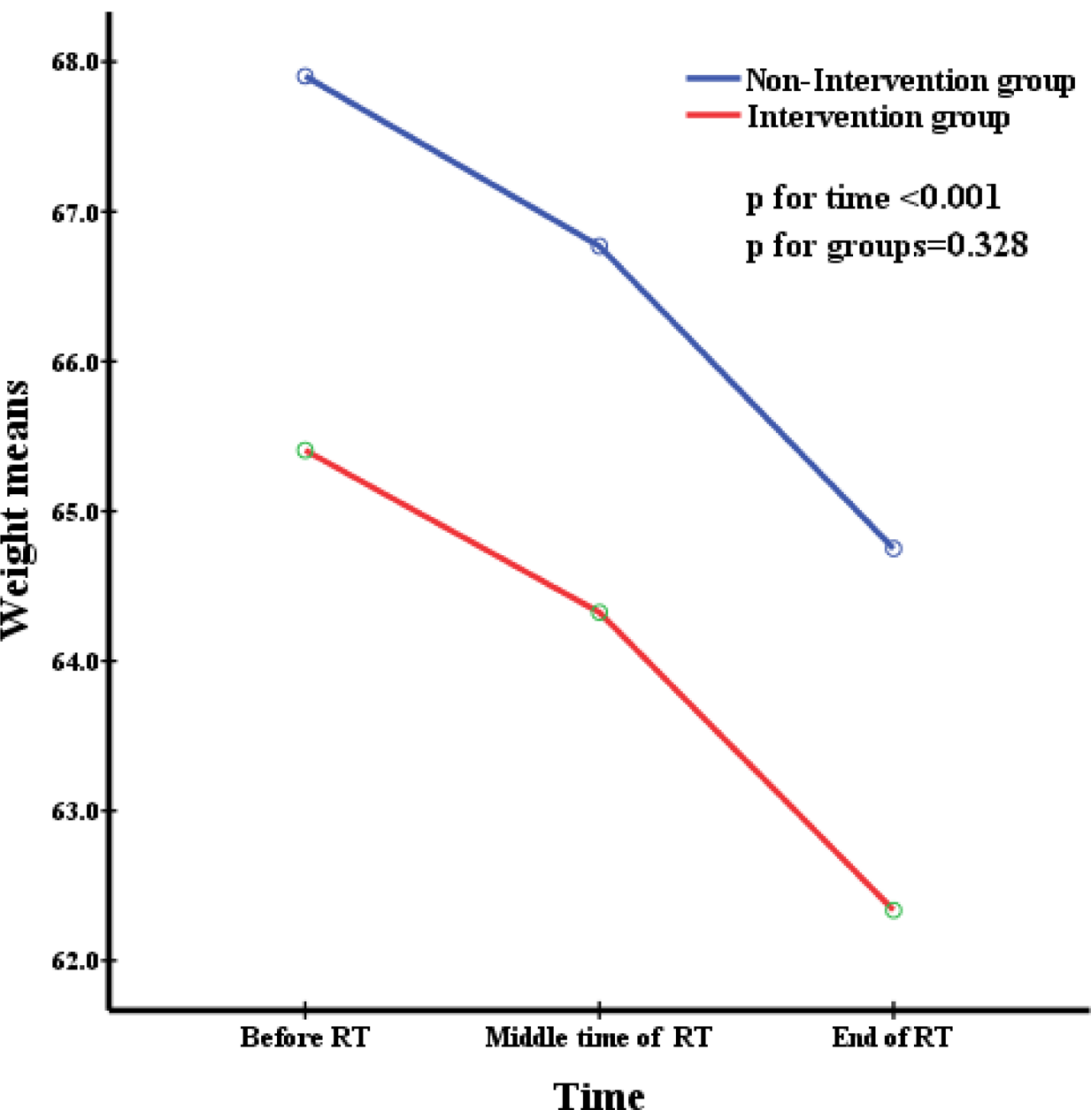
Weight means at different time during radiation therapy

**Figure 2:**
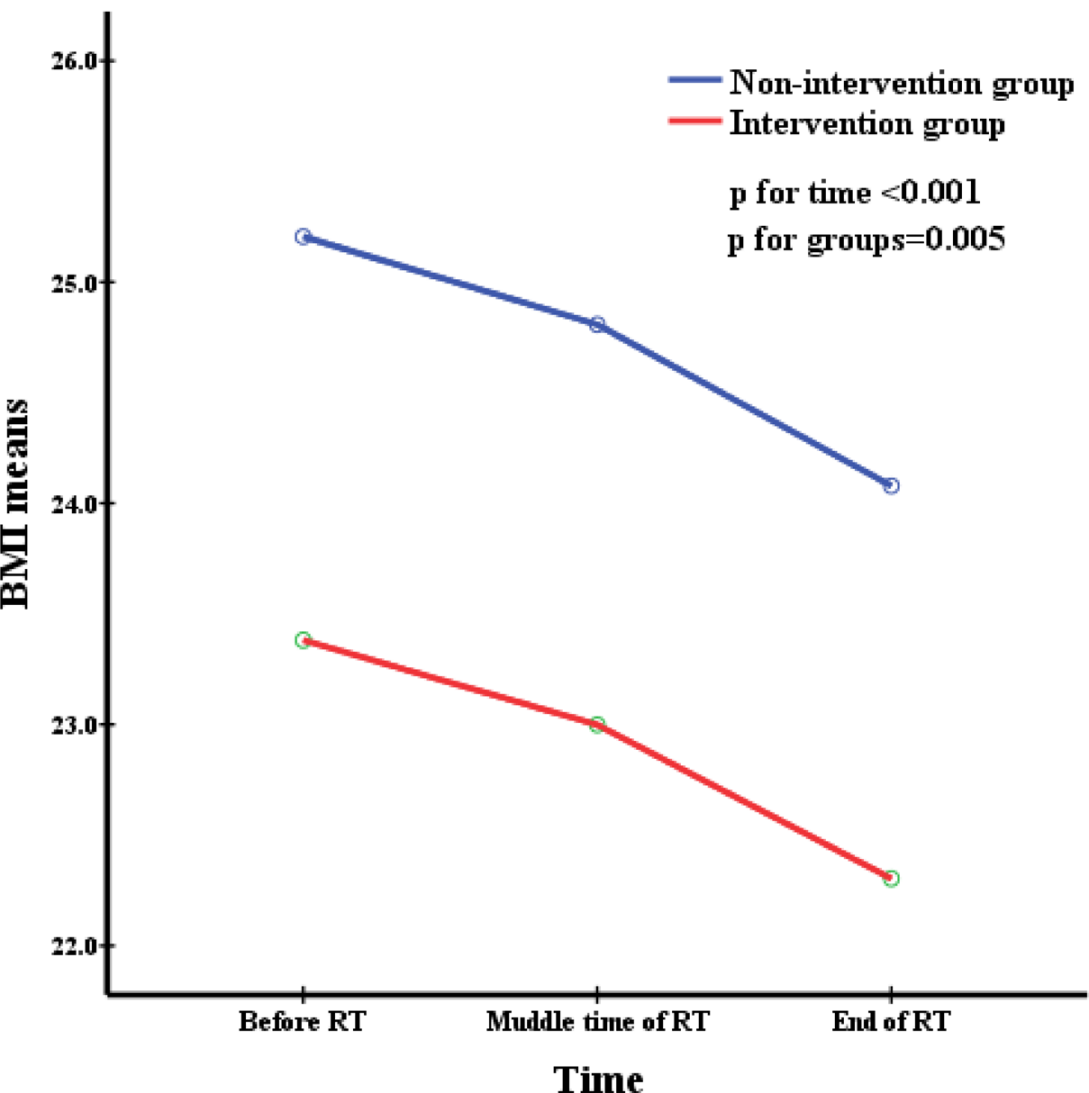
BMI means at different time during radiation therapy

**Figure 3:**
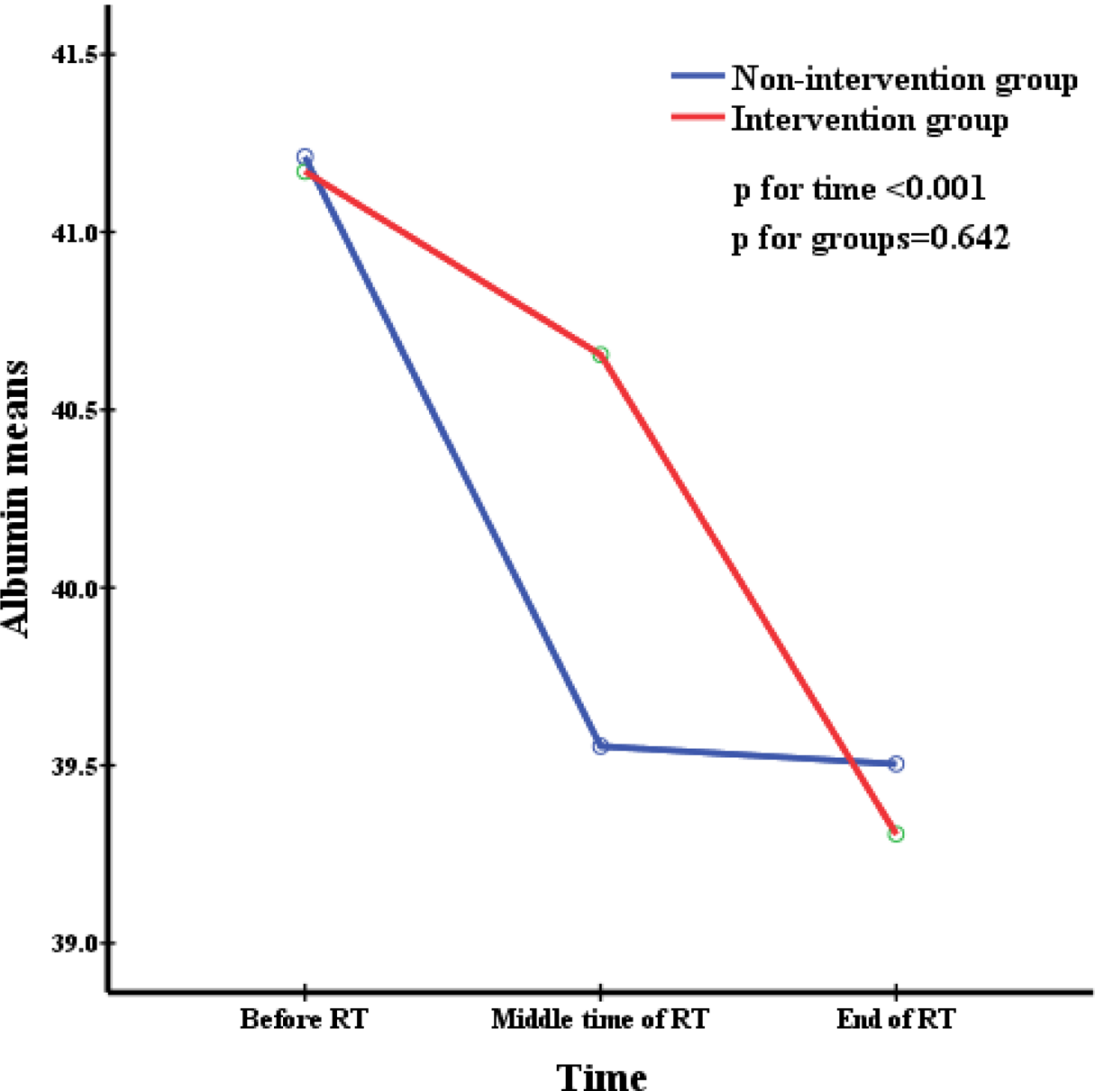
Albumin means at different time during radiation therapy

**Figure 4:**
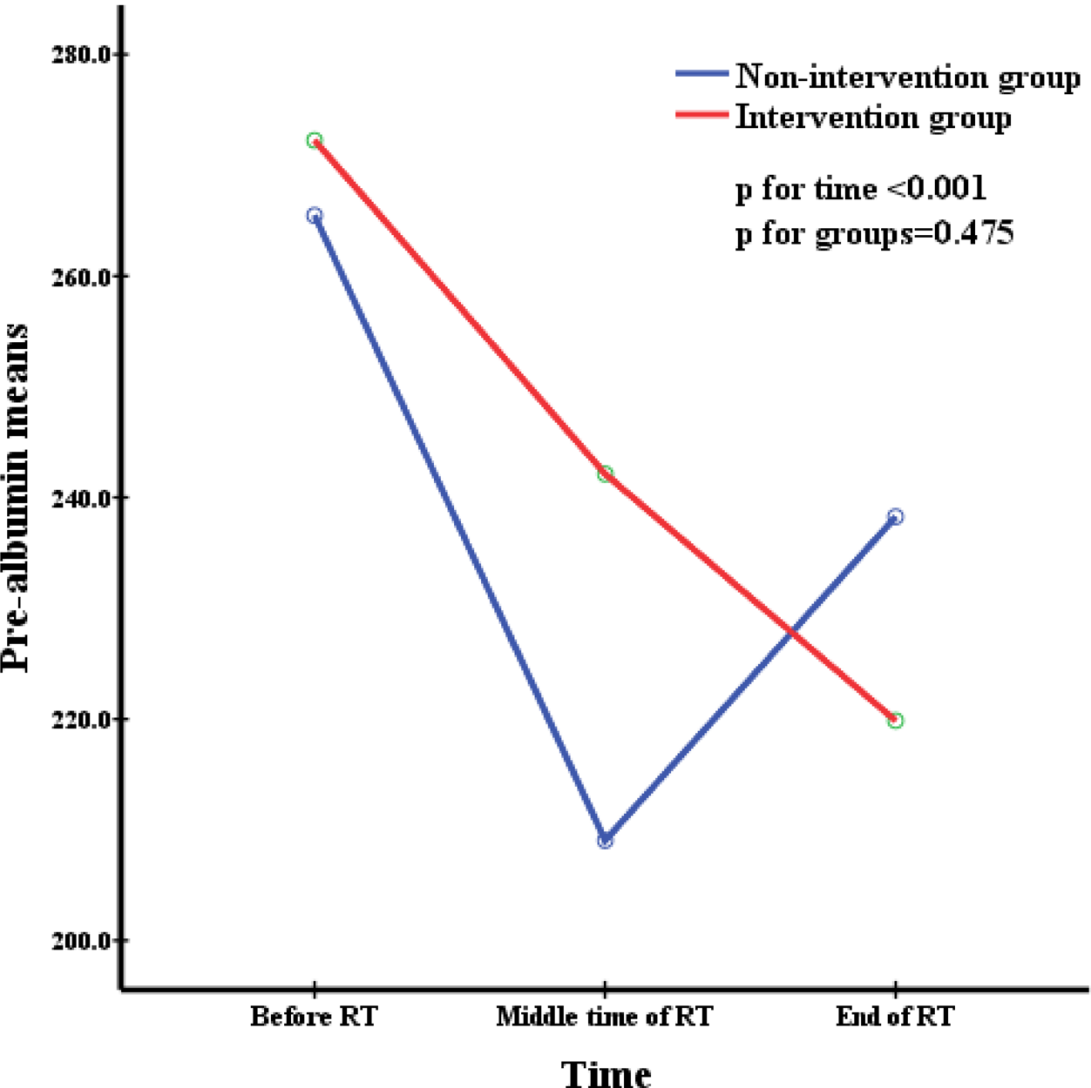
Pre-albumin means at different time during radiation therapy

**Figure 5:**
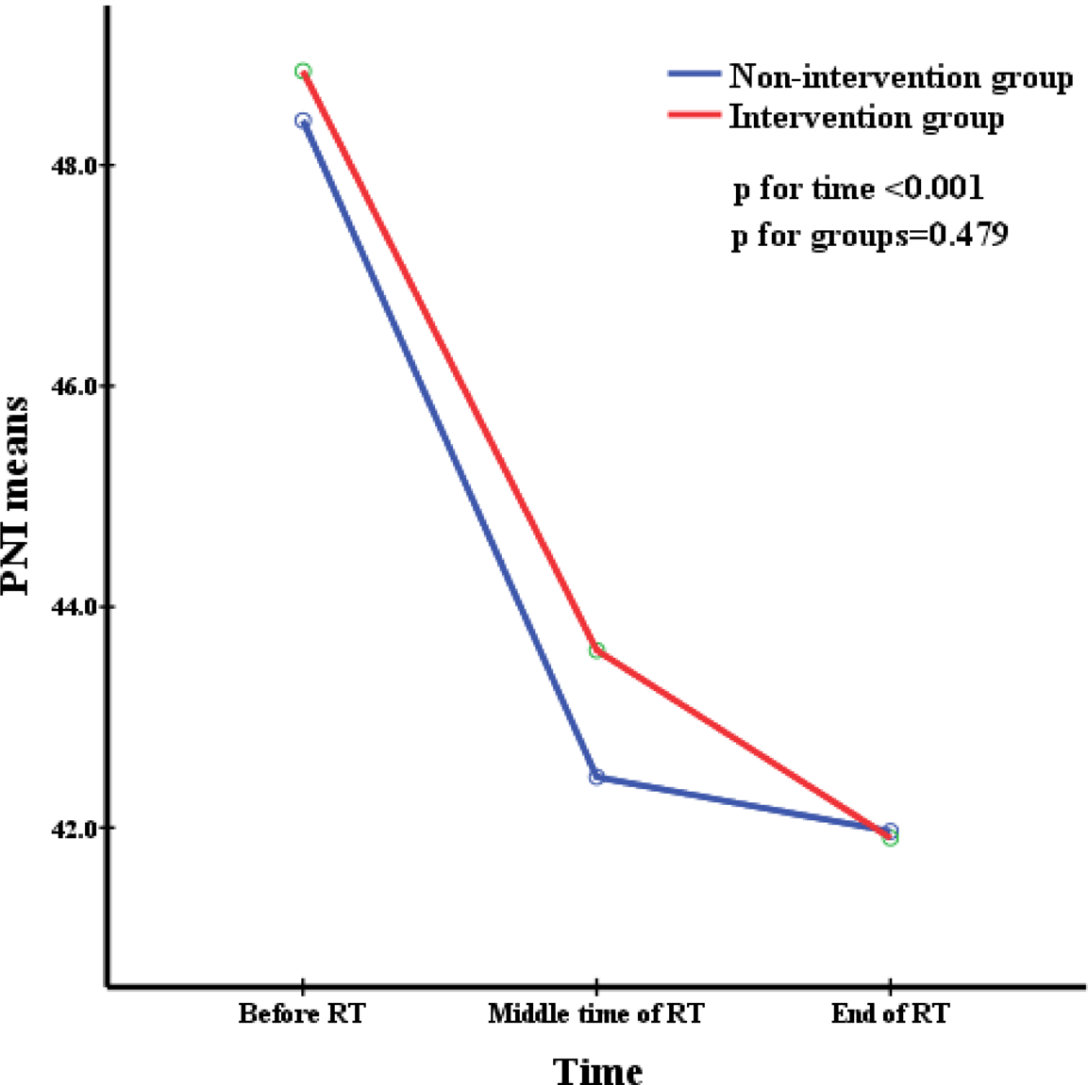
PNI means at different time during radiation therapy

All patients were divided into two groups depending on whether they accepted nutritional support. The repeated measures were used to analyze changes in nutritional indicators before, during, and after radiation therapy and to simultaneously compare the difference in nutritional status between the two groups at the same time point. This was delineated in Figures 1 to 5. All the indicators including weight, body mass index (BMI), albumin and pre-albumin levels, and the prognostic nutritional index dramatically declined in both groups compared with their levels before radiation therapy (All *p* < 0.001). However, no significant difference was found regarding the mean values of the nutritional indicators before, during, and after radiation therapy between the two groups (All *p* > 0.05), except BMI during radiation therapy, which was in a significantly better condition in the non-intervention group at all times.

As summarized in Table 1, all the baseline data were similar between the nutrition intervention group and the control group, except BMI. The BMI at the baseline level and before RT in the non-intervention group was significantly higher than those in the intervention group (*p* = 0.026 and 0.019, respectively). There was no significant difference in the extent of weight loss and mucositis between the two groups (*p* = 0.343 and 0.598, respectively). Nutritional support did not seem to show favor weight maintenance in our study.

When we performed our analysis based on weight loss, we found that weight loss **>** 5% was positively correlated with severe (grade ≥ 3) mucositis. Seventeen percent of the patients suffered from mucositis (grade ≥ 3) in the weight loss **>** 5% group, whereas only 10%, which was much less, suffered from mucositis in the weight loss of ≤ 5% group (*p* = 0.015). The comparison of nutritional parameters, baseline data, stage, treatment, and the adverse reaction of patients in groups were summarized in Table 2. There was no impact of nutrition intervention on weight loss (*p* = 0.343).

Table 3 showed how grade ≥3 radiation-induced oral mucositis was the prognostic factor for poor malnutrition (odds ratio, OR =3 .232, *p* = 0.021, confidence interval, CI [1.198, 8.820]). Besides this, patients with a decrease of **>** 15% in the pre-albumin level was more likely to be malnourished, perhaps pre-albumin can be an ideal bio-marker for malnutrition(OR = 2.442, *p* = 0.041, CI [1.036, 5.757]). Consistent with the results of former analyses, we did not find that current protocols of nutrition intervention can effectively reduce the risk of malnutrition (OR = 1.217, *p* = 0.704, CI [0.042, 3.348]).

**Table 3:** Factors associated with weight loss by logistic regression analysis in 117 patients

Characteristics	OR	95%CI	*p* value
Age	0.965	0.925–1.007	0.100
Basic weight	1.126	0.970–1.306	0.118
Weight before RT	0.886	0.762–1.030	0.115
Sex (Male vs. Female)	2.587	0.838–7.979	0.098
T stage (T3–4 vs. T1–2)	1.149	0.378–3.491	0.807
N stage (N3 vs. N0–2)	0.400	0.093–1.731	0.220
Neo-chemotherapy (Yes vs. No)	0.920	0.040–21.199	0.985
Current chemotherapy (Yes vs. No)	1.209	0.079–18.616	0.892
Radiation technique (IMRT vs. TOMO)	0.536	0.190–1.516	0.240
Intervention (Yes vs. No)	1.007	0.991–1.023	0.418
Mucositis (≥ 3 vs. 0–2)	3.215	1.143–9.046	0.027
PA decrease (**>** 15% vs. ≤ 15%)	2.481	1.038–5.930	0.041

Abbreviations:RT = radiation therapy; PA = pre-albumin; OR = odds ratio; CI = confidence interval.

## DISCUSSION

Nutrition intervention during the treatment of tumor therapy has attracted much attention. Although nutritional support can't directly kill tumor cells, the value of nutritional therapy should not be overlooked. In our study, our objective was to evaluate the effectiveness of nutrition intervention during radiation therapy for patients with locoregionally advanced NPC, observe the change in nutritional indicators, and explore the factors affecting nutritional status. We found that the nutritional care current used in our center can't sufficiently reverse the undernutrition resulting from radiochemotherapy.

Weight, BMI, pre-albumin and albumin levels, and PNI are very common immunological and nutritional indictors. Figures 1 to 5 showed how the nutritional status of patientsgradually declined, and this nutritional deterioration can be seen in both groups. The result was consistent with the study by Kang [32]. Although nutrition intervention was given to patients who were undernourished, no improvement was seen in their nutrition status. The conclusions we derived from the results of our study, chiefly those concerning how current nutritional intervention protocols in our center cannot sufficiently reverse the nutrition situation of those who have nutrition risks during radiation therapy were as follows: firstly, there was no consistent standard nutritional screening criterion, and the nutritional assessment was completed by the consulting physician, which means the results may be subjective. Secondly, because of patients’ low awareness of the importance of nutritional support during tumor therapy, the compliance of many patients in nutrition therapy may be poor, as soon as they feel a little better, they refused continue nutritional support, and it may not enough to reverse the undernutrition status. Metabolism is a continuous process in the human body, with cyclical daily intake and consumption, but the effectiveness of nutritional support is quite short term. Therefore, we need to develop an effective long-term but easily implementable and non-excessive nutrition treatment plan for patients. Lastly,, because this was a retrospective study, the level of BMI was significantly higher in the non-intervention group at the baseline and before RT, as shown in Table 1. It was apparent that the nutritional status was much better in the non-intervention group. Such bias may have resulted in a false null/insignificant effect of the investigated intervention. Once again, the criterion for screening patients who need nutritional support is of great important.

Table 2 suggested that significantly more people suffered from ≥ 3 grade radiation-induced mucositis in a group of patients with weight loss > 5%; hence, we speculated that severe mucositis could lead to a loss of intake and then eventually result in weight loss, and in turn, nutritional insufficiencywas an unfavorable factor for mucous membrane reparation. The logistic regression analysis showed that ≥ 3 grade radiation-induced mucositis and a decrease in pre-albumin level to > 15% were more likely to weight loss. In other words, severe mucositis and a sharp decrease in pre-albumin level can be indicators for those who need nutritional therapy, as shown in Table 3. This finding is consistent with the results of Dilek Unal's study [33].

Our results did not find the advantage of nutritional intervention during radiotherapy; however, we still can't ignore the importance of nutritional intervention in tumor therapy. Insufficient nutrition will affect chemotherapy tolerance [34], influence the level of neutrophils [35], and cause even more severe adverse reactions during therapy, thus preventing patients from accepting radical therapy. It is hypothesized that the undernutrition status is relatively prone to form hypoxic tumor cells and even adverse to re-oxygenation during radiotherapy. Hypoxic tumor cells are insensitive to radiation, and malnutrition may reduce tumor response to radiotherapy, but further study is required to prove this hypothesis. The importance of nutritional care has been proposed in literature but remains controversial. Thus, strong conclusions can't yet be derived; not everyone will benefit from nutrition intervention—how to select patients who need it and how to make an individualized treatment plan are the key problem. Future studies should focus on exploring sensitive screening indicators, effective interventions and the time of intervention. Our center is conducting a clinical trial called “The Impact of Standard Nutritional Intervention in Patients with Locoregionally Advanced NPC who Underwent Neo-Chemotherapy and Concurrent Chemoradiotherapy.” We hope to discover effective screening criterion and positive nutrition intervention protocols that could be offered as personalized adjuvant treatment to improve nutritional status during radiotherapy.

## MATERIALS AND METHODS

### Patients

We conducted a retrospective study, and all 117 cases included in our analysis were treated at Zhejiang Cancer Hospital between December 2015 and March 2016. The inclusion criteria for this study were as follows: 1. Histology: newly diagnosed and histologically-proven undifferentiated or non-keratinizing squamous cell carcinoma of the nasopharynx.2. Stage: III-IVB NPC. All patients were non-disseminated and restaged using the 7th edition of the American Joint Committee on Cancer (AJCC) staging system. Tumor staging was based on routine examinations (physical examination, nasopharyngeal fiberoptic endoscopy, chest X-ray, abdominal sonography, magnetic resonance imaging [MRI], bone scan, positron emission tomography-computed tomography [PET-CT]).3. Treatment modality: Patients treated using radical neo-chemotherapy ± IMRT/ tomography radiation therapy (TOMO) with or without platinum-based concurrent chemotherapy. The study was approved by the institutional review board of Zhejiang Cancer Hospital. As this was a retrospective analysis of routine data, we requested and were granted a waiver of individual informed consent from the ethics committee. The patient records and information were anonymized and de-identified prior to analysis.

### Treatment

All the patients first underwent a pretreatment baseline evaluation including a complete medical history, physical and neurological examinations, hematology and biochemistry profiles, an MRI scan of the nasopharynx and neck, chest and abdominal computed tomography or chest radiography, and abdominal sonography. The treatment plans were determined according to the standard protocols depending on the tumor stage and general health of the patient. All the patients were treated with neoadjuvant chemotherapy plus continuous definitive radiochemotherapy with 70 Gy/32F for the nasopharyngeal region by using a linear accelerator (6–8 MV). The radiation dose range in lymph node-positive areas was 64–70 Gy/32F. In total, 93/117 (79.5%) patients received IMRT, of whom 24 (20.5 %) received TOMO. Every patient in our research was daily asked to weight themselves and tell the physicians about food intake. We gave oral nutritional support (oral nutritious liquids) when weight loss was found or food intake was markedly reduced (decrease roughly to 60%). And we also gave parenteral nutrition when patients underwent mucositis and swallow problems. . No metabolic modulators such as progestins, steroids and possibly eicosapentaenoic acid was used. No routine nutrition support was used during induction chemotherapy. Even it is preferring the enteral route, oral nutritional support and parenteral nutrition (PN) were the main method of nutritional support. As most patients in our research refused to use tube feeding and transnasal / percutaneous routes.

According to research by Scolapio JS [36], the use of PN is recommended if patients present with mucositis or have severe radiation enteritis. Ninety-one patients in total were given nutritional support that included enteral and parenteral nutrition. We did not monitor the diet of the remaining 26 non-nutrition-intervened patients, and their diet was based on their own choice, motivation, and ability.

### Data collection

We gathered data from the medical records that included age, sex, body weight, and height; pre-therapy laboratory counts of white cells, neutrophils, lymphocytes, hemoglobin and platelets; albumin and pre-albumin; TNM classifications, and type of treatment. The data were measured at the baseline, onset of radiation, and at the end of treatment. The prognostic nutritional index(PNI), an indicator of nutritional status and systemic inflammation, was calculated using the following formula: serum albumin (g/L) + 0.005 × total lymphocyte count/Μl [37]. Weight loss during treatment was calculated as follows: (weight at the beginning of radiation) - (weight at last day of radiation).

### Statistical analysis

The data were analyzed using SPSS software (version 20.0; IBM SPSS, Chicago, IL, USA). The distributions of patient characteristics among the groups were assessed using the *t*-test for continuous variables, and chi-square test or Fisher's exact test for categorical variables.

We divided all the patients twice based on different variables. Firstly, the 117 patients were divided into the nutrition intervention group and the control group, depending on whether they accepted nutrition support. Repeated measures were used to analyze changes in nutritional indicators before, during, and after radiation therapy and to simultaneously compare the difference in nutritional status between the two groups at the same time point. Then, the two groups were defined according to the percentage of weight loss: ≤ 5% = non-malnourished group, and weight loss > 5% = malnourished group. Malnutrition was defined as a weight loss of > 5% of the baseline [38]. The chi-square test and logistic regression analysis were used to explore the influence factors for weight loss. Statistical significance was set for two-tailed *P* values < 0.05.

## CONCLUSIONS

Our study showed that the nutritional status of the patients gradually declined during treatment. We concluded that grade ≥ 3 radiation-induced oral mucositis would aggravate the extent of malnutrition during radiation therapy in patients with locoregionally advanced NPC. Pre-albumin was a predictive marker for weight loss in nasopharyngeal carcinoma patients. However, the current nutritional intervention protocols practiced during radiation course cannot significantly reverse the status of nutrition.
